# Procedural Aspects and Physiologic Mechanisms of the Deep Inspiratory Maneuver

**DOI:** 10.1155/2012/961423

**Published:** 2012-04-09

**Authors:** Simon Tilma Vistisen, Peter Juhl-Olsen, Christian Alcaraz Frederiksen, Hans Kirkegaard

**Affiliations:** ^1^Research Centre for Emergency Medicine, Aarhus University Hospitals, Nørrebrogade 42, Building 30, 8000 Aarhus, Denmark; ^2^Department of Anaesthesiology & Intensive Care, Aarhus University Hospitals, Skejby, Denmark

Sir, we have read the paper by S. Preau et al. “*Hemodynamic changes during a deep inspiration maneuver predict fluid responsiveness in spontaneously breathing patients*” with great interest. Based on the fundamental ideas of dynamic hemodynamic monitoring, inducing cardiac preload variations and monitoring corresponding pulse pressure variations [1], the paper presents a novel technique of varying cardiac preload in spontaneously breathing (SB) patients that does not necessitate specialised equipment apart from routine clinical monitoring. The paper is indeed relevant because it is, to our knowledge, the first to present the feasibility of a deep breathing method (deep inspiratory maneuver, DIM) for inducing preload changes. For the purpose of better understanding the presented technique, we wish to pose the following questions for the authors.

1. Concerning compliance and applicability in terms of intention-to-treat: it is not clear which proportion of SB patients with acute circulatory failure was able to comply with the DIM. Can compliance be expected to be as high as 87% (26 of 30) for this generally defined patient group?
2. Concerning the calculations: DIM-induced changes are defined by the authors as.   DIM-induced changes = (maximal value during DIM − minimal value during quiet SB prior to DIM)/((maximal value during DIM − minimal value during quiet SB prior to DIM)/2).
  - How large was the time window when estimating the *minimal value during quiet SB prior to DIM*? Did the time window vary or was it fixed?
  - The *maximal value during DIM* was found in either phase 2 or phase 4 of the DIM. In which phase was the maximal value typically found? Were the maximal values during the two phases generally of the same magnitude?
3. Instructing the patients to perform the DIM: this is important should the technique be validated by other study groups and in turn gain widespread use.
  - Did the authors in any way evaluate if the *slow continuous inspiration strain* was associated with a more or less constant inspiratory flow or was most air generally inspired in the first phase of inspiration, perhaps amplifying maximal value during DIM phase 2? How were instructions given on inspiratory flow rate?
  - Did you make any instructions to the patients (directly or indirectly) what tidal volume should be during DIM? Did they inspire to, for example, *maximal* or *convenient* lung volume?
4. Concerning the physiological mechanisms: did you experience any patients with significant heart rate variability (HRV)? The respiratory part of HRV (with its subsequent varying passive filling time of the ventricles) could be another mechanism inducing preload variations and as such it could confound results obtained with the DIM technique. On the other hand, HRV is known to be significantly reduced during sepsis [2].

We also have a few comments and suggestions to the proposed physiological mechanisms.

The decrease in left ventricular stroke volume during DIM phase 1 is suggested to be induced by increasing ventricular afterload. However, we think that another and perhaps more important mechanism is the reduced intrathoracic pressure reducing left ventricular preload during the initial phase of inspiration—until the increasing right ventricular stroke volume with the delay of pulmonary transit time again increases left ventricular preload substantially. In line with this suggestion, but as a “reversed mechanism,” we also believe that the increasing intrathoracic pressure during passive expiration (DIM phase 4) contributes somewhat to the increasing left ventricular (preload and) stroke volume in DIM phase 4 in addition to the suggested overall increase in intrathoracic blood volume.

As a last comment, we would like to suggest that the DIM phases 2 and 4 could potentially disclose right ventricular failure: a large maximal value during DIM phase 2 relies on biventricular preload responsiveness whereas a large value in DIM phase 4 relies mostly, if not fully, on left ventricular preload responsiveness. Even though the authors did not encounter any cases of preload unresponsiveness associated with high ΔPPdim (specificity was 100%) low phase 2 values associated with high phase 4 values could theoretically disclose right ventricular failure—even when taking interventricular dependence into account.
